# Development and implementation of food and nutrition labelling policies in Mongolia: a policy analysis

**DOI:** 10.1017/S1368980026102390

**Published:** 2026-03-26

**Authors:** Nyamragchaa Chimedtseren, Bridget Kelly, Heather Yeatman, Anne-Therese McMahon, Batjargal Jamiyan

**Affiliations:** 1 School of Medical, Indigenous and Health Sciences, University of Wollongong, Northfield Avenue, Wollongong, NSW 2522, Australia; 2 Nutrition Department, National Centre for Public Health, Mongolia; 3 Independent Scholar

**Keywords:** Food, Nutrition, Label, Food policy, Policy analysis, Consumer

## Abstract

**Objective:**

To analyse food and nutrition labelling policies in Mongolia, with the aim to identify key facilitators and barriers in the policy process and to propose priority actions to address these challenges.

**Design:**

A qualitative study utilising semi-structured individual interviews explored opinions and views of policy stakeholders on Mongolian food and nutrition labelling policies.

**Setting:**

Ulaanbaatar city, Mongolia

**Participants:**

Eighteen policy stakeholders, including government officials, representatives of consumer organisations and food producers.

**Results:**

Food labelling regulations in Mongolia were developed as part of broader reforms of the food system control to respond to changes related to the country’s transition to a market economy. Government leadership, along with technical support from international agencies, facilitated the development of these regulations. Key barriers identified in policy development were industry opposition, lack of consumer engagement, disruptions from government changes and funding shortages. Policy implementation was hindered by delays in operational regulations, inadequate infrastructure and limited knowledge and funding.

**Conclusions:**

To date, the development and implementation of food and nutrition labelling policies in Mongolia have been limited and insufficient. Given the health and nutritional impacts of the nutrition transition, prioritising nutrition labelling policies is essential and should emphasise consumer needs. Key actions should include the establishment of clear regulations, active stakeholder engagement, well-resourced implementation, capacity building among regulators and producers, and consumer education.

The WHO recommends nutrition labelling as an effective strategy to promote healthy diets by providing consumers with information on the nutritional content of food and supporting informed decision-making. This approach helps to combat non-communicable diseases (NCD) among other strategies that improve food environments.^(1)^ The Codex Alimentarius Commission (Codex) under the FAO of the United Nations and the WHO develops international food standards to promote public health and facilitate international trade. The Codex Guideline on Nutrition Labelling (2021) mandates the declaration of energy, protein, fat, carbohydrates, saturated fat, sodium/salt and total sugars on all pre-packaged foods.^(2)^ Supplementary nutrition labelling, such as front-of-pack labelling (FOPL), is optional and varies by country. Globally, implementation of nutrient declarations has increasing in recent years, through both mandatory or voluntary measures.^(3)^

Most research on food and nutrition labelling policies has focused on high and upper middle-income countries.^(4,5)^ In contrast, progress on such policies in low- and middle-income countries (LMIC) remains less understood.^(6,7)^ Limited evidence suggests that policy development in LMICs is often driven by champions within government and shaped by funding availability.^(8–13)^ This contrasts with the policy dynamics in high-income countries (HIC), where consumer demand for nutrition labels and public health advocacy play a larger role.^(14)^ In LMICs, barriers to policy implementation included weak governance, limited regulatory and industry expertise, funding constraints and industry resistance.^(10,11,15)^

Mongolia is an emerging economy that transitioned from socialism to democracy in the early 1990s. The country is undergoing significant reforms across all sectors, including the food system, and is experiencing a rapid nutrition transition.^(16,17)^ In recent decades, Mongolia’s overweight and obesity rates have surged, affecting half of the population aged 15–69 years in 2019.^(18)^ The country has a high burden of NCDs, with CVD, cancer and digestive system diseases, constituting 63·1 % of deaths in 2022.^(19)^

The Mongolian government has adopted policies addressing food labelling, including the Food Safety Law (2012), ^(20)^ the amended Food Law (2012), ^(21)^ the food labelling standard MNS 6648–2016 on requirements for the labelling of food products ^(22)^ and a voluntary FOPL guideline (2017).^(23)^ The Food Safety Law, along with the food labelling standard, serves as the primary legislation governing food labelling, setting requirements for mandatory label information.^(20)^

The Food Safety Law (2012) regulates food safety across the food chain, including production, processing, storage, distribution, sale, import, packaging and labelling. It contains a dedicated chapter on labelling, which mandates that information be clear, truthful and non-deceptive, and provided in Mongolian, English or Russian, including details on nutritional content.^(20)^ The amended Food Law (2012), the overarching legislation on food, aims to ensure a sustainable supply of high-quality, nutritious and safe food. It includes labelling provisions that emphasise accuracy and clarity as core principles of food safety.^(21)^ Although labelling requirements were initially drafted under the Food Law, they were later incorporated into the Food Safety Law during the

review process.^(24)^

The food labelling standard MNS 6648–2016 was based on Codex standards and came into force in January 2018. It mandates the declaration of energy, fat, carbohydrates, protein, saturated fat, sugar, salt and any nutrients related to nutrition or health claims.^(22)^

In addition, a voluntary FOPL guideline,^(23)^ adopted by the Health Minister’s Order in 2017, introduced a traffic light colour-coding system, which indicates per-serving levels of energy, saturated fat, sugar and salt, categorising them as ‘high’, ‘medium’ or ‘low’, along with their percentage contributions to the Mongolian dietary reference intakes.

There has been limited research on the development and implementation processes of these policies in Mongolia. This study examined Mongolia’s food and nutrition labelling policies, aiming to identify key facilitators and barriers in the policy process and to propose priority actions to address these challenges.

## Methods

### Study design and sampling

A qualitative study was undertaken in Ulaanbaatar, the capital city of Mongolia, between November 2017 and March 2018. Opinions and views regarding food labelling policy and regulations in Mongolia were explored via semi-structured individual interviews with government officials from food and health organisations, representatives of consumer organisations and food producers.

A purposive sampling strategy ensured representation of key stakeholders relevant to food and nutrition policy. Key national-level government organisations, including Ministry of Health (MoH), Ministry of Food and Agriculture (MoFA), State Specialized Inspection Agency (SSIA), City Specialized Inspection Agency, Mongolian Agency for Standardization and Metrology (MASM), and the National Centre for Public Health (NCPH), were targeted. In addition, two leading national non-governmental organisations (NGO), representing consumer organisations and food producers, were included. From these organisations, thirteen officials holding top and mid-level positions, along with two other individuals who were part of working groups on food laws development, were identified as potential key informants and invited to participate in the study.

Additionally, a desk review was conducted to analyse documents and information related to food and nutrition labelling policy in Mongolia, aiming to identify the existing regulatory framework and contextualise findings from the stakeholder interviews.

### Development of the interview guide

A semi-structured interview guide was developed using Walt & Gilson’s Policy Analysis Triangle Framework (PATF),^(25)^ which examines how policy context, processes and actors influence policymaking, policy content and implementation, highlighting factors that contribute to policy success or failure.^(26,27)^ This framework has been used in previous studies on nutrition labelling policies in LMIC.^(8)^

The guide included open-ended questions on Mongolia’s food labelling policies, their development and implementation, the roles of policy actors, policy effectiveness in assisting consumer choices, and suggestions for improvement (Appendix 1). The guide was refined following two pilot interviews with public health professionals from the NCPH, Mongolia, to ensure face validity in terms of content, clarity and acceptability.^(28)^ Content validity was assessed by public health experts from the University of Wollongong, Australia.

### Data collection

Of the fifteen individuals contacted, two declined participation, citing work commitments or insufficient knowledge in the area. Once interviews commenced, additional participants were recruited based on recommendations from key informants. In total, eighteen key informants were interviewed, representing government agencies (*n=*8), consumer organisations (*n=*2), food producers (*n=*1) and policy working groups (*n=*7). Government organisations held prominent representation in the sample, reflecting their pivotal role in developing and implementing food policies. The lead researcher, experienced in conducting in-depth interviews, conducted the sessions in Mongolian, either face-to-face or via video call. The interviews were audio recorded, transcribed verbatim and translated into English. Each interview lasted 40 to 60 min.

For the desk review, official policy documents were sourced from government websites and supplemented by searches of relevant governmental and international organisation websites, as well as Google web searches. A total of twenty-five official documents, including resolutions, orders, decrees, parliamentary meeting transcripts, and reports, were identified and analysed, alongside several online news articles.

### Data analysis

The policy processes of Mongolia’s food and nutrition labelling policies were analysed using three theoretical frameworks: the PATF, Sabatier’s Advocacy Coalition Framework (ACF) and Kingdon’s Multiple Streams Framework (MSF). The ACF examines how advocacy coalitions, policy actors’ beliefs, and both stable and dynamic external factors influence policy change through resources and constraints.^(29)^ The MSF explains policy agenda setting through the convergence of three streams: problem, political and policy. For an issue to reach the agenda, all streams must align, with shifts in the problem or political stream creating a window of opportunity that policy entrepreneurs can leverage to advance the issue.^(30)^

In our analysis, the PATF was used to identify and describe existing policies and processes across the full policy cycle, including context, agenda setting, development and implementation. The ACF was applied to examine the entire policy process, focusing on coalition formation, belief systems, power dynamics, and strategies influencing policy change. The MSF was used specifically for the agenda setting and policy development stages, providing insights into how food safety and labelling issues entered the agenda through the interaction of problems, policy solutions and political opportunities. This integrated approach enabled a comprehensive understanding of the policy processes.

The transcripts were thematically analysed using NVivo Version 12 (Plus) by QSR International Pty Ltd. (2018). Prior to coding, the lead researcher carefully reviewed the transcripts and developed preliminary analytical notes. Inductive content coding in NVivo was used to identify initial codes, which were refined through iterative analysis and constant comparison.^(31)^ Codes were subsequently grouped into themes and sub-themes, which were collectively discussed and agreed upon by the research team. The sub-themes were organised according to the domains of the PATF – policy context, content, process and actors, and further analysed through the lenses of the ACF and MSF (Table 1).

**Table 1.** Integrated policy analysis of Mongolian food and nutrition labelling policy

	Theme	Sub-theme	Definition of sub-theme	Policy Analysis Triangle Framework	Advocacy Coalition Framework	Multiple Streams Framework
1	Historical socio-political context	Food system changes	Reflects the changes in the country’s food system driven by the transition.	**Context:** Structural and economic transitions have driven changes in the food system.	The socio-political transition served as an external factor for policy change.	–
		Diminished food system control	Weak institutional capacity and an insufficient regulatory framework constrained the management of the increased food imports and domestic food production during the transition period.	**Context:** Weakness in food system governance hindered the effective regulation of the food system during the transition period.		**Problem stream:** • Inadequate food quality and safety control underscored the need for policy reform and a strengthened food system control. • Concerns about food safety have gained public and political attention influencing the national mood. Widespread distrust in the food supply, combined with weak enforcement, has created pressure for change. • Frequent foodborne disease outbreaks influenced the national mood and raised political concern, as they were attributed to weakened food safety standards.
		Reduced food safety and quality	Deterioration of food safety and quality standards for domestic products, along with inadequately regulated imports, resulted from a weakened food system control.	**Context:** Declined food safety standards, unregulated imports, and widespread distrust in food safety and quality		
		Food safety incidents	Refers to the emerging risks of foodborne diseases.	Frequent foodborne disease outbreaks have drawn public and political attention, highlighting the importance of food safety.		
2	Perceived policy progress	Significant progress	Adoption of the food laws and the food labelling standard represents a significant progress in food labelling policies. In particular, the inclusion of labelling provisions in the Food Safety Law is marked as a critical milestone.	**Content:** The food laws and the food labelling standard provide the regulatory framework for food and nutrition labelling.	–	–
		Novel policy	Food labelling is a relatively new policy in Mongolia.	**Content:** Food labelling policies represented a new policy area that emerged in response to the transition.	–	–
		Not meeting consumer needs	The labelling standard fails to meet consumer needs, permitting the use of foreign languages for labelling and requiring complex information that is difficult for consumers to interpret.	**Content:** Concerns were raised regarding the effectiveness of food labelling policies in meeting consumer needs.	The lack of strong coalition representation has limited consumers’ influence on policy development.	**Problem stream:** Consumer issues struggled to reach the agenda, resulting in policy gaps due to limited consumer influence.
		Lack of regulations in specific areas	Refers to the gaps in the labelling standard, where certain aspects of food labelling are not adequately addressed.	**Content:** Gaps in the labelling standard left some areas unregulated.	–	–
3	Agenda setting	Adoption of food labelling under broader food safety policies	Refers to the integration of food labelling into the primary food safety law, rather than its adoption as a standalone policy.	Food labelling was introduced as part of a broader food safety policy framework.	–	–
		Pivotal food safety incident	The methanol-taint vodka incident, which claimed 11 lives, triggered a public health emergency, bringing the food safety issue to the government’s attention and prompting a crisis-driven policy response.	The incident prompted the government to respond with increased urgency.	The external event triggered policy change and the mobilisation of coalitions.	**Problem stream:** The focusing event increased pressure on regulators and created a policy window in which the problem, policy and political streams aligned, advancing the food safety issue onto the government agenda.
		Need to strengthen food control and enhance the regulatory framework	Highlights the inability of existing regulations to adapt to the evolving food system and underscores the need to strengthen food safety control.	The government acknowledged the need to strengthen food control and the regulatory framework.	The government coalition was mobilised to respond to the issue.	**Political stream:** The government responded to the external event by acknowledging the need to enhance the food system control.
		Align national regulations with international standards	Refers to the alignment of the labelling policies with internationally benchmarked frameworks in food safety and food labelling, including World Trade Organisation agreements, Codex standards, and European Union regulations. A decentralised, risk-based approach to food safety that emphases a transition from exclusive government oversight to shared responsibility across the food chain.	International guidelines, including Codex standards and regulations from other countries, were used as benchmarks for guidance.	External factors, including international guidelines and benchmark policies, have influenced the adoption of food safety and labelling policies.	**Policy stream:** Policy proposals were informed by international benchmark policies to address existing gaps in the system.
		Need for labelling regulations for both imported and domestic products	Refers to the need to regulate labels of imported products, including addressing foreign language use and inconsistent information, as well as labels of domestic products, to prevent food fraud and ensure ingredient transparency, including the disclosure of food additives.	Consumers have raised concerns about product labels written in foreign languages, the sale of expired products, and the lack of transparency in the declaration of food additives. Inspection agencies have frequently reported labelling violations.	The government coalition was mobilised to respond to the issue.	**Problem stream:** • Consumer confusion over foreign language food labels, along with the concerns about transparency of label information and expired products, underscored the need for stronger regulation. • Frequent non-compliances with the labelling standard highlighted the regulatory gaps. • Despite the growing burden of obesity and NCDs, there was limited concern or demand for nutrition labelling from both the government and consumers.
4·1	Policy development (The Food Law and the Food Safety Law)	Leadership of government	Highlights the government’s leadership in formulating the food laws.	Key policy actors, included government agencies, public health professionals, industry representatives and international organisations.	The government coalition comprised of various government agencies, international organisations and members of parliament. It supported amendments to the Food Law and the development of the Food Safety Law. The coalition’s core beliefs focused on protecting public health, while its policy core beliefs prioritised truthful and accurate labelling, including the provision of expiry dates, disclosure of food additives, and the use of Mongolian language labels for imported products.	**Political stream:** • New government leadership was pivotal in advancing the issue both to the agenda and the adoption stage. The adoption of the food laws was influenced by political timing, with the laws ultimately enacted by the newly selected government • The government played the role of policy entrepreneurs in advancing the Food Law by linking the problem, policy solutions, and political opportunities.
		Engagement and support of international organisations	Technical expertise and funding support of the IFC played a major role in the development of the Food Safety Law.	International organisations, particularly the IFC played a supporting role in developing food safety and food labelling policies by providing technical support and funding assistance.	International organisations were key stakeholders within the government coalition.	The IFC played the role of policy entrepreneurs in advancing the Food Safety Law by linking the problem, policy solutions, and political opportunities.
		Disruptions due to government changes and funding limitations	Government changes and funding shortage delayed the development of the food laws.	Government changes have slowed policy development.	Policy change was delayed due to institutional constraints, including frequent government turnovers.	**Political stream:** Government changes hindered the policy development.
		Food safety prioritised over nutrition labelling	Refers to regulators’ strong focus on food safety and low priority given to nutrition labelling.	Regulators prioritised food safety, giving less emphasis on food and nutrition labelling.	**Contextual impact on belief systems of advocacy coalitions:** The government coalition’s core beliefs were influenced by the rising concerns over food safety.	**Problem stream:** The limited knowledge and expertise among regulators regarding food and nutrition labelling contributed to the lack of emphasis on nutrition labelling within the food labelling policies.
		Regulators’ limited knowledge and expertise in food and nutrition labelling	Refers to regulators’ limited knowledge and expertise in food and nutrition labelling.	**Contextual impact on actors:** Regulators lacked expertise in food labelling, which influenced their perspectives on the policies.	**Contextual impact on coalition resources:** The government coalition had limited knowledge and expertise in food and nutrition labelling.	
		Industry opposition	Refers to industry opposition to requiring product labels exclusively in Mongolian.	Industry opposition to Mongolian-only labelling led to a compromise permitting Russian and English labels.	The industry coalition, which included industry representatives and some members of parliament, held core beliefs favouring minimal regulatory intervention to protect commercial interests. The coaliton’s policy core beliefs were about opposing the requirement for label information on imported products to be provided exclusively in Mongolian.	**Political stream:** Industry lobbying influenced government decisions.
4·2	Policy development (The food labelling standard and the FOPL guideline)	Leadership of public health professionals	Highlights the role of public health professionals in developing the food labelling standard and FOPL guideline.	• Identified key policy actors, including government agencies, public health professionals, industry representatives and international organisations. • Food labelling policies were mainly developed by public health experts in government agencies.	The public health coalition comprising, of government health and standardisation agencies held core beliefs of protecting public health and promoting healthier food choices, and policy beliefs of implementing mandatory nutrient declarations and mandatory FOPL policy.	**Political stream:** Public health professionals played the role of policy entrepreneurs in developing the food labelling standard and the FOPL guideline.
		Funding support of international organisations	Funding support from the WHO played a major role in the development of the food labelling standard and FOPL guideline.	The WHO supported the development of food labelling policies by providing funding.	International organisations were key stakeholders in both the government and public health coalitions.	**–**
		Industry engagement and opposition	Refers to industry’s active involvement in the development of the food labelling standard, as well as its opposition to mandatory FOPL policy.	Strong industry opposition to mandatory FOPL policy influenced policymakers to adopt a voluntary approach instead.	The industry coalition prioritised commercial interests and held policy core beliefs of favouring voluntary FOPL policy instead of mandatory FOPL.	**Political stream:** Industry lobbying influenced government decisions.
4·3	Policy development (Consumer engagement in policy development)	Lack of consumer engagement and public awareness campaigns	Refers to limited consumer engagement in policy process, particularly low public demand for food and nutrition labelling, and the lack of awareness campaigns on the proposed regulations.	Consumers were less informed and underrepresented in policy discussions.	Weak public mobilisation in coalition formation. Consumers were not seen as key stakeholders.	**Political stream:** Consumers’ lack of information and limited engagement in the policy process led to the lack of consumer demand for policy change.
		Consumers’ limited knowledge and understanding of food and nutrition labelling	Refers to consumers’ limited knowledge and understanding of food labelling, especially nutrition labelling.	**Contextual impact on actors:** Limited engagement with food labelling under the previous regime left consumers both less informed and less knowledgeable about it.	Consumers had limited power of advocacy within the coalition structure.	**Problem stream:** Consumers’ limited knowledge and understanding of food and nutrition labelling likely resulted in the lack of demand for improved labelling.
5	Policy implementation	Frequent labelling violations	Refers to frequent non-compliances with the Food Safety Law and the previous labelling standard (At the time of the study, the new standard had not yet been enforced).	Enforcement challenges for the labelling policy	–	–
		Delay in follow-up regulations	Refers to the significant gap between the adoption of the law and the food labelling standard, highlighting the lack of enforcement mechanism.	**Context:** The delay in developing the food labelling standard has resulted in the absence of established enforcement mechanisms.	Institutional constraints have resulted in the absence of enforcement mechanisms, delaying the enforcement.	–
		Inadequate infrastructure and limited resources	Refers to weak enforcement capacity due to inadequate infrastructure, including limited laboratories, lack of monitoring and evaluation, and insufficient training, as well as limited knowledge, expertise and resources.	Weak enforcement of the labelling policy stemmed from insufficient regulatory infrastructure and limited resources in both expertise and funding.	Limited resources in knowledge, expertise and funding constrained the policy implementation.	–
		Challenges faced by industry in complying with the new standard	Refers to technical barriers to complying with the new standard, including nutrient testing capacity, label update costs, and existing printed stock.	Enforcement of the labelling standard was postponed by one year to provide the industry with the sufficient time to comply with the new regulation.	The industry coalition delayed the enforcement of the new labelling standard by one year.	–
		Minimal use of food labels by consumers	Refers to consumers’ limited use of food labels, which is focused mainly in checking expiry dates.	The limited use of food labels by consumers is likely to undermine the effectiveness of the policy.	The limited use of food labels by consumers is likely to undermine the effectiveness of the policy.	–
		Need for consumer nutrition education	Highlights the need for complementary policies, including public education campaigns, to improve consumer awareness of food labels.	Need for complementary nutrition education interventions.	• –	–

FOPL, front-of-pack labelling; IFC, International Finance Corporation.

## Results

The integrated policy analysis, guided by the PATF, ACF and MSF, identified five overarching themes: *‘historical socio-political context’*, *‘perceived policy progress’*, *‘agenda setting’*, *‘policy development’* and *‘policy implementation’*, comprising multiple sub-themes (Table 1). These sub-themes, analysed through the three frameworks, provided a comprehensive understanding of the food and nutrition labelling policy processes.

### Historical socio-political context

The analysis of the policy context revealed that Mongolia’s historical socio-political context has been a key driver shaping current food safety and labelling policies. The sub-themes *‘food system changes’*, *‘diminished food system control’* and *‘reduced food safety and quality’* reflected the impacts of the country’s transition from a socialist to a market economy. This shift led to the collapse of state-owned food industries, the emergence of a private sector, trade liberalisation and a surge in food imports. Participants highlighted weaknesses in the capacity to manage decentralised food production and food imports, which contributed to reduced food safety and quality standards.

‘Following the enactment of the 1999 Food Law, shifts in the economic and political environment led to increased private sector involvement in food production, along with significant challenges in regulating individual food imports’. (Regulator)

The deterioration in food safety and quality emerged as a critical issue requiring policy attention. The sub-theme *‘food safety incidents’* referred to frequent outbreaks of foodborne diseases during this period, linked to the decline in food safety. Public and political concerns intensified in response to these repeated outbreaks, fostering widespread distrust in the national food supply and increasing pressure to reform food safety policy and strengthen the food control system. According to the MSF, this situation falls within the problem stream (Table 1).

### Perceived policy progress

Participants identified the Food Safety Law and the Food Law as the primary legislation governing food and nutrition labelling, with the food labelling standard serving as the key guideline. Most participants regarded the adoption of these laws and the standard as representing *‘significant progress.’* In particular, the inclusion of labelling provisions in the Food Safety Law was viewed as a critical milestone, reflecting increasing attention to the issue.

‘In 2012, the Food Safety Law was enacted, the first comprehensive legalisation of food product labelling in Mongolia …This represented a substantial regulation’. (Regulator)

‘To my understanding, there were no regulations governing labels before… (Member, Law development working group)

Food labelling was perceived by participants as a *‘novel policy’* issue with limited relevance under the previous economic regime, due to the lack of diversity in food products, particularly processed foods. This contrasts with the long history of food labelling regulations in many other countries.^(12,32)^

The sub-theme *‘not meeting consumer needs’* highlighted shortcomings in current food labelling policies in addressing consumer needs. Two key issues emerged: the complexity of mandatory label information, which often exceeds consumers’ nutrition literacy, and the use of multiple languages on labels. According to the ACF, this policy gap stemmed from weak consumer representation within policy coalitions, resulting in limited public involvement in policy processes (Table 1). One participant noted on the disconnect between labelling requirements and consumer capabilities.

‘The latest labelling standard has weak ties to the nutrition education levels of its end users, with the requirements set too high. The volume and complexity of nutrition information may be difficult for the general public to understand and use in practice’. (Regulator)

Other participants expressed concerns about the permitted use of multiple languages on food labels, noting that this approach fails to serve consumers unfamiliar with foreign languages. As one participant pointed out:

‘Nearly 70 to 80 percent of the population does not understand Russian or English, yet almost 80 to 90 % of processed products are imported products [with labels written in these languages].’ (Member, Law development working group)

Additional concerns were captured under the sub-theme *‘lack of regulations in specific areas.’* Participants highlighted poor legibility, inconsistent content and formatting, and questioned the credibility of label information due to the absence of a verification system. A desk review of the food labelling standard confirmed several gaps, including the lack of clear regulations on nutrition and health claims, the absence of requirements for the legibility of nutrition information, and missing labelling standards for imported products.

### Agenda setting

The sub-theme *‘adoption of food labelling under broader food safety policies’* reflects the integration of food labelling into broader food safety policy, driven by regulatory reforms aimed to address changes in the food system and establish the regulations and infrastructure necessary for a safe, high-quality food supply. Accordingly, this study examined the policy processes of the amended Food Law and Food Safety Law.

According to the MSF, increasing public and political concern over food safety, driven by weakened food system control and frequent foodborne disease outbreaks, contributed to a prevailing national distrust in the food supply, signalling the ripeness of the problem stream (Table 1). A *‘pivotal food safety incident’* involving methanol-tainted vodka, which claimed 11 lives in 2007, exposed weaknesses in food system control and moved food safety onto the government’s policy agenda.

In response, the government acknowledged *‘the need to strengthen food control and enhance the regulatory framework.’* ^(33)^ It also recognised the limitations of the 1999 Food Law in addressing the challenges of an evolving food system^(33)^ and subsequently prioritised its amendment in key strategic plans.^(34,35)^ This shift was facilitated by a favourable political climate following the formation of a new government, enabling the government coalition to advance the issue on the policy agenda.

To better address emerging demands within the food system and ‘*align national regulations with international standards’*, Mongolia adopted a decentralised, risk-based food safety approach^(36)^ and drew on internationally benchmarked frameworks in developing its food policies (Table 1).

‘When amending the law, we looked at international standards such as Codex, as well as food laws and food safety regulations from other countries, including the EU.’ (Member, Law development working group)

Consumer complaints about unclear foreign language labels, undisclosed food additives and expired products have driven growing demand for stricter labelling regulations (the sub-theme *‘need for labelling regulations for both imported and domestic products’*). Frequent non-compliance with existing standards further underscored the urgency of implementing stronger regulations.

‘Numerous consumer complaints about unsafe imported foods, expired dates, and confusing labels led us to integrate these concerns into the law [Food Safety Law].’ (Regulator)

However, most participants, except those directly involved in drafting the labelling standard and the FOPL guideline, did not view labelling regulations as a strategy to promote informed food choices or to address obesity and NCD.

### Policy development

The *‘policy development’* theme comprised three components – development of the food laws, development of the labelling standard and FOPL guideline, and consumer engagement – under which eleven sub-themes reflecting facilitators and barriers to the policy process were identified (Table 1).

#### Development of the Food Law and the Food Safety Law

According to the ACF, the food labelling policy subsystem emerged around the development of the Food Law and the Food Safety Law. Two main advocacy coalitions were identified: the government coalition, comprising health, food and agriculture agencies, inspection and standardisation bodies, international organisations and some members of parliament, and the industry coalition, consisting of food producers, industry associations and some members of parliament. These coalitions influenced the policy process in line with their core and policy core beliefs (Table 1).

The government’s initiative was a key facilitator in the development of the Food Law, reflected in the sub-theme *‘leadership of government.’* A working group under the MoFA led the amendment, with MoFA officials acting as policy entrepreneurs. The initiative began around 2007–2008 but faced *‘disruptions due to government changes and funding limitations’*, delaying its adoption until 2012.

‘The working group for the Food Law amendment included representatives from food and health-related government organisations, NGOs and international organisations.’ (Member, Law development working group)

The initial draft of the Food Safety Law was prepared with technical and financial support from the International Finance Corporation (IFC), a World Bank Group organisation focused on private sector development, as part of its Reforming Business Inspection project launched in 2008.^(37)^ External consultants working with the IFC assisted in drafting the law. The sub-theme *‘engagement and support of international organisations’* highlighted the IFC’s role as a key policy entrepreneur.

In 2010, the MoFA led a joint working group to refine and harmonise the drafts of two laws. The group included representatives from key government agencies (MoFA, MoH, SSIA, NCPH, MASM and the General Customs Authority), as well as NGOs and international organisations, such as the IFC, WHO and FAO.

By 2011, the drafts were submitted to Parliament, with the amended Food Law submitted by the government, and the Food Safety Law by a parliamentary group. Following further parliamentary discussions, both laws were approved in December 2012. Political timing likely influenced their adoption, as they were passed by the newly established government.

The sub-theme *‘food safety prioritised over nutrition labelling’* reflects participants’ perspectives on food and nutrition labelling. Although participants acknowledged the importance of food labelling, discussions were often redirected toward broader food safety concerns, highlighting gaps in existing standards and governance. The reluctance to engage in discussions on labelling issues may reflect participants’ *‘limited knowledge and expertise in food and nutrition labelling.’*

The industry coalition’s opposition led to the rejection of the working group’s initial proposal during parliamentary discussions, resulting in a compromise that permitted English and Russian to be used as additional label languages alongside Mongolian (the sub-theme ‘*industry opposition’*).

‘…For example, should we ask the producer to provide label information for Haribo candy imported from Germany in Mongolian just because of this requirement? We need to stop such unrealistic things.’ ‘I would like to keep the requirement for Mongolian labelling but also add English or Russian, since these languages are taught in secondary schools, allowing the information to be provided in one of them. This is ultimately about protecting people’s right to know what they consume…’ (Meeting minutes from the Parliamentary session)^(24)^

#### Development of the food labelling standard and the FOPL guideline

The food labelling standard was developed by the government as an operational regulation of the Food Safety Law. The standard was adapted from the amended Codex standards for food and nutrition labelling and was developed with the financial support from the WHO (the sub-theme *‘funding support of international organisations’*). The development of the FOPL guideline was included in the Ministry of Health’s strategic plan, and its design was informed by international models, including the UK traffic light labelling system.

Based on the ACF, two advocacy coalitions emerged within the nutrition labelling policy subsystem, centred on the development of the food labelling standard and the FOPL system: the public health coalition and the industry coalition. The public health coalition supported mandatory nutrient declarations and simplified nutrition labelling to promote healthier food choices. Conversely, the industry coalition advocated for a voluntary FOPL system, reflecting its policy core beliefs favouring minimal regulatory intervention (Table 1).

The MoH led the development of both the food labelling standard (2016) and the FOPL guideline (2017), with public health professionals from the NCPH and MASM acting as key policy entrepreneurs (the sub-theme *‘leadership of public health professionals’)*. Drafts were circulated among stakeholders, including government agencies, consumer organisations, NGOs and food producers. Although the process was government-led, the food industry was actively involved and opposed mandatory FOPL, citing trade barrier concerns (the sub-theme *‘Industry engagement and opposition’*). This led to a compromise, resulting in the adoption of a voluntary FOPL system.

‘The MoH and NCPH made strong efforts to include a clause on FOPL in the new standard. However, the idea faced strong opposition from almost everyone, particularly food producers.’ (Academic)

‘The Food Producers Association, which has over 350 member organisations across 18 aimags [provinces], gathered and submitted feedback from its members.’ (Representative of Food producers’ NGO)

#### Consumer engagement in policy development

In both the food labelling and nutrition labelling policy subsystems, consumers possessed limited advocacy power and had minimal engagement in policy processes. Although consumer organisations were occasionally included in working groups, there were no public hearings or advocacy campaigns on the proposed regulations (the food laws, the food labelling standard and the FOPL guideline), and opportunities for consumers to participate in policy processes were largely limited to providing feedback on government websites (the sub-theme: *lack of consumer engagement and public awareness campaigns*). According to participants, consumers generally have *‘limited knowledge and understanding of food and nutrition labelling’*, which likely contributed to their low demand for improved labelling (Table 1).

‘We do not have principles of tailoring [regulations] to each consumer. Consumers were not included in the working group responsible for the labelling standard. To be honest, in the discussions, only one or two NGOs and food producers were included, but not consumers. (Regulator)

### Policy implementation

Six sub-themes were identified under the *‘policy implementation’* theme (Table 1). These reflected both facilitators and barriers to the policy implementation.

As the new standard had not yet been implemented at the time of the study, participants were asked to comment on the implementation of the Food Safety Law and the previous food labelling standard. They widely agreed that enforcement of these policies was weak, citing *‘frequent labelling violations’* reported by inspection agencies. Participants also noted that food companies often prioritised packaging and label design over the label content.

‘Reports from inspection agencies indicate prevalent labelling violations, with estimated non-compliance rates ranging between 30 and 40 percent.’ (Regulator)

Key barriers to implementation cited by participants included *‘delays in follow-up regulations’* and *‘inadequate infrastructure and limited resources.’* Although the Food Safety Law was enacted in 2012, the food labelling standard only came into effect in January 2018, resulting in a prolonged delay in establishing enforcement mechanisms.

Infrastructure-related issues affecting policy implementation included the limited authority of inspectors, weak legal penalties, insufficient training for both inspectors and food producers, a lack of nutrient testing laboratories, particularly for small manufacturers, and inadequate monitoring and evaluation.

‘…the failure to enforce the law is due to insufficient legal fines and penalties.’ (Regulator)

‘To comply with the new standard, it is necessary to list all the ingredients and nutritional content. The challenge is whether the laboratories have the capacity to meet these requirements. Not every factory has the resources.’ (Regulator)

In addition to inadequate infrastructure, limited resources in terms of knowledge, expertise and funding hindered the government’s capacity to enforce regulations, promote policies, raise awareness and train stakeholders.

Food producers often lacked the technical knowledge and capacity to meet labelling requirements (the sub-theme *‘challenges faced by industry in complying with the new standard’*). These challenges, combined with the costs of updating labels, contributed to the introduction of a one-year transition period prior to the enforcement of the new standard.

Furthermore, most participants reported that consumers made *‘minimal use of food labels’*, focusing mainly on expiry dates, and attributed this to limited knowledge and understanding of labelling among consumers. They emphasised *‘the need for consumer nutrition education’* to raise awareness of food and nutrition labelling.

‘It seems to me that consumers don’t pay much attention to it [nutrition label] because the general population is poorly educated on nutrition.’ (Regulator)

## Discussion

This study presents the first in-depth analysis of Mongolia’s food and nutrition labelling policy development and implementation, identifying key barriers and facilitators in the policy process. Over the past decade, the Mongolian government has advanced labelling regulations addressing label language, food additive disclosure and expiry dates, measures shaped by the country’s transition to a market-driven economy.

Government leadership, supported by international technical and financial assistance, played a crucial role in developing Mongolia’s food labelling policies, consistent with trends observed in other LMIC.^(8–11)^ Nonetheless, policy progress has faced significant barriers, including industry opposition to mandatory FOPL, limited consumer engagement, and disruptions due to government changes and funding constraints. Industry opposition had a notable impact on the outcome of the FOPL policy, aligning with findings in both LMICs^(8,12,13)^ and in HICs. Consumer participation in the policy process was minimal, consistent with Mongolia’s past decision-making traditions and a weak civil society, contrasting with international practices where public engagement contributed to more effective policy outcomes^(9,12,13,38–41)^.

While Mongolia’s food labelling policies appear to align with Codex standards, major implementation gaps persist due to infrastructure and resource limitations. These challenges are rooted in the country’s rapid transition to a market economy, which outpaced regulatory responses and left the government, industry and consumers unprepared to implement and comply with new policies. Similar implementation issues have been reported in other research on nutrition and health claims in Mongolia.^(42)^

Despite the growing burden of obesity and NCDs in the country, there was limited concern or demand for nutrition labelling from both the government and consumers. This contrasts with countries where such policies are considered central to promoting healthy diets.^(8,11,13,14,43)^ Although nutrition is recognised as a policy priority by the Mongolian government, reflected in the National Nutrition Program, nutrition labelling has remained a minor focus, embedded only within broader food environment policies. This lack of emphasis has hindered policy integration, a challenge commonly observed in other LMICs.^(8,10,11,15,44)^ Conversely, in jurisdictions with strong policy values and actor beliefs, the implementation of labelling policies has been more effective.^(43,45)^

The findings highlight the need for directive government leadership to counteract the negative impacts of food system changes and to promote informed food choices. With heavy reliance on imported processed foods, Mongolia faces accelerated risks of unhealthy diets, obesity and NCDs,^(16)^ similar to other developing countries undergoing nutrition transition. Addressing these challenges requires a comprehensive suite of structural food environment policies such as those regulating food marketing, pricing, promotion, provision and retail rather than relying solely on individual-level policies such as public education.

In integrating food labelling into broader nutrition strategies, Mongolia can build on its success with previous food environment interventions, such as salt reduction initiatives, which achieved positive outcomes through a coordinated approach involving policy, public education, industry collaboration, and WHO support.^(46)^ International evidence indicates that successful FOPL implementation relies on a combination of strong political will, government leadership and active engagement from civil society, including consumer groups, academia, health professionals and the media. Effective policy reforms are grounded in scientific evidence, stakeholder consultations, transparency and consumer awareness campaigns, and should be implemented as government regulations rather than voluntary guidelines.^(47)^

In Mongolia, civil society actors, including public health academics, consumer organisations and NGOs, can serve as policy entrepreneurs to elevate nutrition labelling on the policy agenda. By advocating for stronger labelling regulations and highlighting the link between food labels, dietary choices and health outcomes, they can mobilise support and influence policy coalitions. Priority actions should include mandating FOPL, requiring labels in the Mongolian language, strengthening regulatory infrastructure, improving consumer label awareness and understanding, and fostering stakeholder participation in policy processes, particularly from consumers. The government should seek continued international support from organisations such as the WHO and FAO for technical assistance, capacity building and policy guidance in implementing the national food and nutrition labelling policy. Given that current labelling policies have not been updated, an evaluation of their implementation is recommended to inform future improvements.

## Limitations of the study

The study focused on the viewpoints of regulators and national policy stakeholders, which may have skewed findings towards the government’s perspective, potentially overlooking the viewpoints of food producers and consumers. Future research should explore these perspectives for more comprehensive understanding. Additionally, the study primarily included national-level key informants, possibly missing challenges at the sub-national level. Extending future research to local levels would help to capture grassroots policy implementation. However, our sample included all relevant agencies and senior key positions, with snowball sampling identifying additional relevant individuals, ensuring that the findings represent the food labelling policy process at the national level.

## Conclusions

The evolution of Mongolia’s food and nutrition labelling policy stemmed from the need to adapt the food system control during the country’s transition to a market economy. Despite government efforts, integrating Codex standards into national policies faced challenges rooted in the previous regime’s context. Industry opposition, limited knowledge and resources, inadequate infrastructure and the lack of consumer engagement have hindered policy processes. To mitigate the adverse health impacts of the nutrition transition, it is crucial to implement nutrition labelling policies alongside other strategies that foster healthy food environments. Government actions should focus on establishing clear regulations, engaging stakeholders actively, ensuring well-resourced implementation, building capacity among regulators and producers, and promoting consumer education.

## Appendix 1. Key informant interview guide

**Opening commentary**

I would like to hear your thoughts on food labelling policy and regulations in Mongolia. I am interested in how this policy was developed and what have influenced the introduction of this policy and what was the participation consumers in the development and implementation of the policy. Also I would like to know your opinions regarding the effectiveness of the policy itself and its implementation. There are no right and wrong answers.

**Question 1:** In relation to policy/ies (XX) for which your section has responsibility, can you please describe the role of this policy in food labelling regulation/policy matters?

**Question 2:** Can you please describe for me how this policy was developed and what were the key drivers for developing such a regulation/policy?

Prompts:

• Can you please tell me more about the key people involved? Or key policy objectives that you were trying to achieve?

• Can you please tell me about key external factors/activities that influenced the development of this policy (e.g. new Codex regulation, key trade partnership agreement, etc.)

**Question 3:** In some countries, governments have been influenced by consumers or the public health communities to design food label information in particular ways. Can you please tell me about how the needs of consumers or of public health have influenced the food labelling regulation/policy processes or decisions?

Prompt:

• Can you please tell me the ways that consumers were consulted on the policy during its development? If not, why not?

**Question 4:** In your opinion, how effective the implementation of this policy in practice? What are the barriers in its effective implementation?

Prompts:

• What are the weaknesses and gaps in the policy?

• Obstacles faced in its implementation

• Implementation by food industries in practice?

• Monitoring and evaluation?

• Barriers from consumer side?

**Question 5:** How do you think that consumers make of use food label information in practice? In your opinion, how helpful is the food labelling policy in assisting consumers to use food labels?

Prompts:

• Using food labels by consumers to assess food safety, comparing products or judging about nutritional quality of a product

**Question 6**: What actions should be undertaken in order to enable consumers make informed food choices by using food labels?

Prompts:

• Policy changes?

• Consumer education?

• Improving of labelling practices?
